# Modeling antecedents of electronic medical record system implementation success in low-resource setting hospitals

**DOI:** 10.1186/s12911-015-0192-0

**Published:** 2015-08-01

**Authors:** Binyam Tilahun, Fleur Fritz

**Affiliations:** Institute of Medical Informatics, University of Münster, Albert-Schweitzer-Campus 1, Gebäude A11, Münster, D-48149 Germany

**Keywords:** Electronic health record, Low-resource setting, D&M model, Computer literacy EMR implementation science

## Abstract

**Background:**

With the increasing implementation of Electronic Medical Record Systems (EMR) in developing countries, there is a growing need to identify antecedents of EMR success to measure and predict the level of adoption before costly implementation. However, less evidence is available about EMR success in the context of low-resource setting implementations. Therefore, this study aims to fill this gap by examining the constructs and relationships of the widely used DeLone and MacLean (D&M) information system success model to determine whether it can be applied to measure EMR success in those settings.

**Methods:**

A quantitative cross sectional study design using self-administered questionnaires was used to collect data from 384 health professionals working in five governmental hospitals in Ethiopia. The hospitals use a comprehensive EMR system since three years. Descriptive and structural equation modeling methods were applied to describe and validate the extent of relationship of constructs and mediating effects.

**Results:**

The findings of the structural equation modeling shows that system quality has significant influence on EMR use (β = 0.32, P < 0.05) and user satisfaction (β = 0.53, P < 0.01); information quality has significant influence on EMR use (β = 0.44, P < 0.05) and user satisfaction (β = 0.48, P < 0.01) and service quality has strong significant influence on EMR use (β = 0.36, P < 0.05) and user satisfaction (β = 0.56, P < 0.01). User satisfaction has significant influence on EMR use (β = 0.41, P < 0.05) but the effect of EMR use on user satisfaction was not significant. Both EMR use and user satisfaction have significant influence on perceived net-benefit (β = 0.31, P < 0.01; β = 0.60, P < 0.01), respectively. Additionally, computer literacy was found to be a mediating factor in the relationship between service quality and EMR use (P < 0.05) as well as user satisfaction (P < 0.01). Among all the constructs, user satisfaction showed the strongest effect on perceived net-benefit of health professionals.

**Conclusion:**

EMR implementers and managers in developing countries are in urgent need of implementation models to design proper implementation strategies. In this study, the constructs and relationships depicted in the updated D&M model were found to be applicable to assess the success of EMR in low resource settings. Additionally, computer literacy was found to be a mediating factor in EMR use and user satisfaction of health professionals. Hence, EMR implementers and managers in those settings should give priority in improving service quality of the hospitals like technical support and infrastructure; providing continuous basic computer trainings to health professionals; and give attention to the system and information quality of the systems they want to implement.

## Background

Implementation of health information systems (HIS) in low-resource settings is not about modernizing healthcare but about saving lives [1]. With the already severe lack of health professionals and basic facilities, especially in remote areas, poor handling of medical documentation is an additional burden. To improve the situation, many international and humanitarian agencies are investing a huge amount of money to improve HIS in those settings. The WHO outlines in its Third Global Forum on Human Resources for Health [2] that getting timely and proper patient information will help utilizing the scarce human and financial resources properly. Because of those initiatives, there is a huge investment in implementing telemedicine, mobile health, electronic medical records and district HIS in those countries, mostly funded by donor organizations. Since implementing and expanding a system usually involves considerable human and financial investment, there is a growing need to identify and prioritize the essential elements for the proper adoption and success of the respective systems [3–6].

A trial-and-error approach of EMR implementation is very costly especially for low-resource settings. There is a need to develop models based on previous implementation experiences or validate existing frameworks for their applicability to predict EMR success. These can be used by managers as an input for prior planning [7, 8]. Several empirical studies in different domains have been conducted to explore this confusing but yet important issue [9, 10]. However, there is still no clear answer of which constructs best measure information system (IS) success. The most used and validated IS success model was developed by DeLone and McLean (D&M) in 1992 and revised in 2003 [11, 12]. The D&M model has been tested and validated in hundreds of studies in the IS domain over the last 20 years [12]. It was specifically validated for its applicability in different domains, such as web portals [13], e-government systems [14] and knowledge management systems [15].

A much smaller body of research has attempted to assess whether the D&M constructs can be applied to HIS success [16–18]. Van der Meijden in his review paper used this model to categorize the different measures of success [19] and found evidence supporting all constructs of the D&M model. Booth et al. also conducted a review of nurses using Information and Communication Technology (ICT) based on the D&M model and found the model to be effective in synthesizing basic elements of health ICT use [16].

In the context of low-resource settings, Hanmer et al. [18] developed a conceptual case study model of factors that affect HIS success by including some of the D&M constructs. Nunes et al. [20] also validated partial components of the D&M model to predict the intention to use for a HIS among health professionals in Brazil. Despite the above studies that use part of the D&M constructs in EMR evaluation, we do not know of any study that brings together all the measures of D&M constructs and validates them for its applicability in assessing EMR success in the context of low-resource settings.

Additionally, most evaluation and case studies of EMR implementations in low-resource settings reported that computer literacy seems to be a main hindering factor for the success of EMR [21–24] especially in a relationship between service quality and IS use [20] but this effect is not yet rigorously tested in those settings.

This study is therefore intended to address these gaps by assessing the validity of the D&M model and by determining the effect of computer literacy on EMR use and user satisfaction among health professionals in a low-resource setting. The main objectives of this study are to:

1. Validate the applicability of the revised D&M model for EMR success evaluations in low resource setting implementations.
2. Assess the effect of health professional’s computer literacy on the relation between service quality and EMR use as well as between service quality and user satisfaction.

## Methods

### Study design

An institutional based quantitative cross sectional study design was chosen, including questionnaires among health professionals of five public hospitals in Ethiopia.

### Theoretical background

**Fig. 1:**
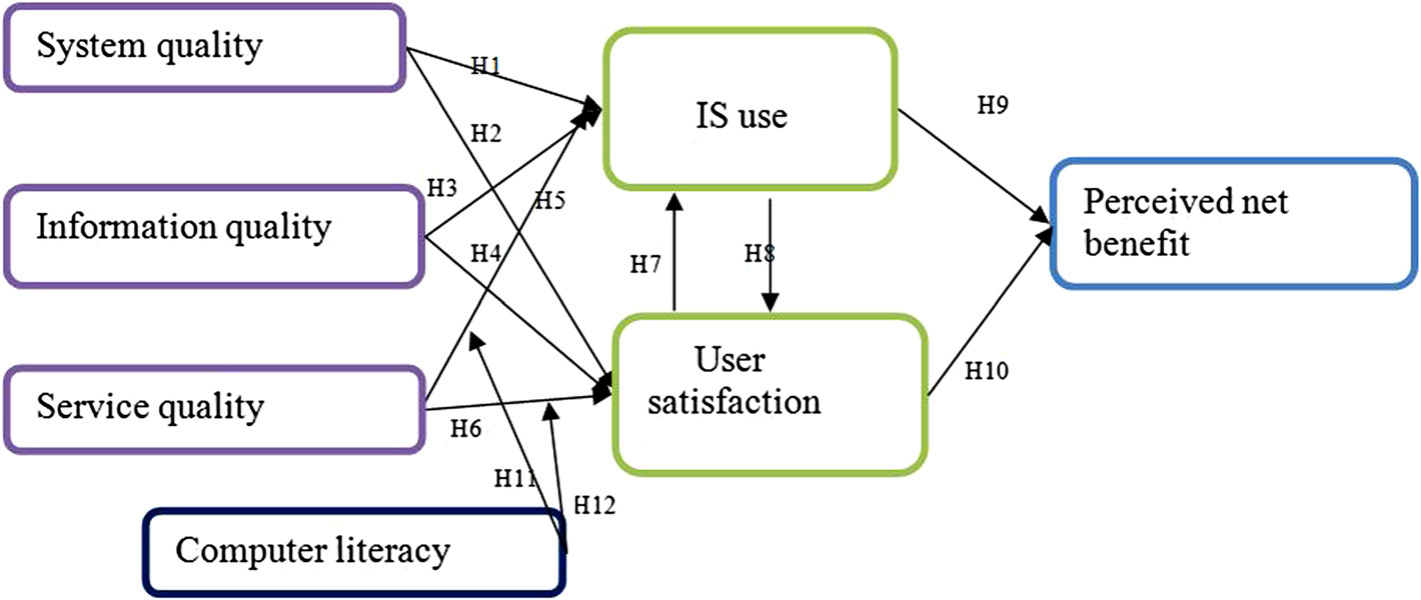
Constructs and hypothesis (H1-H12) of the updated D&M model and the mediating effect of computer literacy in the relationship between service quality and EMR use as well as between service quality and user satisfaction

DeLone and Mclean [12] comprehensively review different IS success measures and propose a model that includes six dimensions for IS success measurement: information quality, service quality, use, intention to use, user satisfaction and net-benefits. The model was validated in hundreds of studies in different domains especially in e-commerce [16]. In this study we hypothesized that the constructs and relationships depicted in the updated D&M model are valid to measure the success of EMR system implementations in low-resource settings. The proposed research framework, research variables, and their relationships are shown in the following Fig. 1 and our hypotheses are explained below (Fig. 1).

#### System quality

System quality addresses whether a system has the required functionality by its user to support the work in question, and ease of use is the most common measure of system quality validated by different researchers [25]. System reliability, response time, user friendliness and stability are additional determinant factors proposed by many researchers [17, 26–28]. With respect to this background this study tests the following hypotheses:

- ***H1: System quality will have a positive effect on IS use.***
- ***H2: System quality will have a positive effect on user satisfaction.***

#### Information quality

Information quality concerns with measures of IS output. Most validated measures of information quality contain perceived usefulness, accuracy, format and timeliness [29, 30]. This study tests the following hypotheses:

- ***H3: Information quality will have a positive effect on EMR use.***
- ***H4: Information quality will have a positive effect on user satisfaction.***

#### Service quality

Service quality addresses the available user support, both internally and externally as well as the additional infrastructures that support the proper adoption of the EMR. Typical measures of service quality include the internal and external support [30, 31]. Hence, the hypotheses to be tested are:

- ***H5: Service quality will have a positive effect on EMR use.***
- ***H6: Service quality will have a positive effect on user satisfaction.***

#### System use and user satisfaction

User satisfaction has been widely used as a surrogate for measuring system success but system use is applied rarely because it is context dependent and difficult to measure. Most IS are mandatory and researchers claim that assessing use in such environments is useless. They rather prefer using perceived usefulness. But just like the system we test in this study, there are those that are used voluntarily. Hence, we use “EMR use” as a measure. We also believe that this is a good opportunity to validate the effect of use on user satisfaction and perceived net-benefit under a voluntarily used system.

For net-benefit, there has been little consensus on how it should be measured objectively. It is usually measured by the perceptions of those who use the IS [32]. Similarly, “perceived net-benefits” is adopted as an important surrogate of EMR success in this study. The hypotheses to be tested therefore are:

- ***H7: EMR use will have a positive effect on user satisfaction.***
- ***H8: User satisfaction will have a positive effect on EMR use.***
- ***H9: EMR use will have a positive effect on perceived net-benefit.***
- ***H10: User satisfaction will have a positive effect on perceived net-benefit.***

#### Mediating effect of computer literacy

Computer literacy refers to the knowledge and skills that enable individuals to use computers effectively for a specific task. Most evaluation and case studies of EMR implementations in low resource settings reported that computer literacy is a main hindering factor for the success of EMR [21–24]. Nunes et al. outlines that computer aptitude is a significant mediating factor in the relationship between service quality and user satisfaction. With mediator we mean a variable that causes mediation in the dependent and the independent variables. Statistically it is to measure the extent of how computer literacy affects the relationship between system quality and EMR use. Agreeing with Nunes et al. hypotheses, we want to extend it by testing the following hypotheses:

- ***H11: User computer literacy mediates the relationship between service quality and EMR use.***
- ***H12: User computer literacy mediates the relationship between service quality and user satisfaction.***

### The EMR system and study setting

The data for this study was collected at five public hospitals in Ethiopia that all use a comprehensive EMR system since three years. The system in use is called SmartCare. It is a nationally scalable EMR system designed specifically for low resource, disconnected settings. It is currently in use in East African countries including Zambia and Ethiopia [33].

### Sampling and participants

The sample size was calculated assuming a 95 % confidence level and 10 % nonresponse rate, a margin of error of 5 % and 10 % contingency, which resulted in 384 participants. The participants of this study were health professionals across all five study hospitals including physicians, nurses, pharmacists and laboratory technologists. The health information technicians were perceived to be good users of the system and they were excluded to avoid bias. The participants were selected by a simple random sampling technique among the respective health professional categories. Ethical clearance was obtained from the University of Gondar Ethical committee and individual consent was obtained from all participants.

### Instrument development and validation

A questionnaire was developed to test the hypotheses. The questions are divided into two categories. The first category contains 15 questions about general socio-demographic data, computer training and current use of the EMR system. The questions were adapted from Mahmood et al. [34], Lawrence & Low [35] and Igbara & Nachman [36]. The second category, consisting of 24 items, was designed to measure the perceived system quality, information quality, service quality, satisfaction, expectation towards future benefits and mediation effects of computer literacy. For the latter the questions were adapted from Nunes et a.l [20], the others from Seddon et al. [37] and Doll et al. [38]. A pretest of the questionnaire was conducted in a hospital other than the study hospitals. Five physicians, eight nurses and three laboratory technology staffs participated and following their perspective, two of the questions were amended in their wording.

Before analysis, we conducted three tests to determine the reliability of the instrument constructs in a single instrument including the reliability of items, composite reliability (CR), and Cronbach’s alpha of constructs. The results of the three tests demonstrated scores for all the items over the criterion as shown in Table 1. Thus, the indicators measuring the constructs in the present study all carried sufficient item reliability, as shown in the following Table 1.

**Table 1:** Evaluation of the measurement constructs reliability

Variable	Item	Abbr	SD	CR	AVE	C α
System Quality	SmartCare is easy to use	SQ1	0.89	0.84	0.68	0.84
	SmartCare is user friendly	SQ2	0.76			
	I find it easy to get SmartCare to do what I want it to do	SQ3	0.71			
	The response time of SmartCare is acceptable	SQ4	0.88			
Information quality	SmartCare provides sufficient information to enable you to do your tasks	IQ1	0.73	0.91	0.74	0.89
	I am satisfied with the accuracy of SmartCare	IQ2	0.86			
	SmartCare generate complete report	IQ3	0.84			
	With SmartCare, I am able to access the information I need in time	IQ4	0.91			
	The reports from other departments are in the format of my need.	IQ5	0.83			
Service Quality	SmartCare is dependable	SQ1	0.86	0.89	0.73	0.85
	My supervisor has been helpful in the use of SmartCare.	SQ2	0.87			
	The available user guides and help function is helpful	SQ3	0.79			
	I can access computers in the ward when I need them	SQ4	0.91			
	The generator automatically backs up power losses	SQ5	0.87			
	The support from Tulane is timely.	SQ6	0.77			
	The reported bugs on the software get fixed in acceptable time frame	SQ&	0.74			
Use	I frequently use SmartCare for my tasks	Use1	0.88	0.91	0.76	0.91
	I am dependent on SmartCare for my task	Use2	0.93			
User Satisfaction	I can finish my task faster with SmartCare	US1	0.89	0.89	0.69	0.86
	SmartCare improves my productivity	US2	0.81			
	SmartCare has positive impact on quality of my task	US3	0.78			
	Overall I am satisfied with SmartCare	US4	0.81			
Perceived net-benefit	I expect SmartCare to make the patient care faster	PN1	0.95	0.95	0.79	0.84
	I expect SmartCare to increase my effectiveness	PN2	0.93			
	I expect SmartCare to make the hospital service better	PN3	0.91			
Computer literacy	I am interested in working with computers	CA1	0.94	0.96	0.81	0.91
	I have moderate skill in using computers	CA2	0.93			
	I take computer trainings in the hospital	CA3	0.89			
	I am playful in technology	CA4	0.91			
	I feel that using computers will support me to be more efficient in the future	CA5	0.96			

SD = Standard loading, CR = Composite reliability C α = Cronbach’s α AVE = Average variance extracted

### Method for data analysis

The SPSS 22.0 statistical software packages were used for descriptive data analysis. AMOS 22.0 structural equation modeling (SEM) was used to clarify the extent of relationships between variables.. The SEM is a powerful statistical method that quantifies and clarifies the extent of relationships between variables. For this reason, SEM was used to test the hypotheses.

This study also used six common model-fit measures to assess the model’s overall goodness of fit, including Chisquare ratio (<3), goodness of fit index (GFI > .9), adjusted goodness of fit index (AGFI > .8), normal fit index (NFI > .9) and root mean square of standardized residual (RMSR < .08) to evaluate overall model fitness. As shown in Table 1, all the model-fit indices exceeded their respective common acceptance levels suggested by previous research [39], thus demonstrating that the measurement model exhibited a fairly good fit with the data collected (*χ*2/d.f. = 2.39, GFI =0 .92, AGFI =0 .87, NFI = 0.92, RMSR = .056). Other validation studies of the D&M model also used this conventional cut of point [13, 14].

## Results

### Socio demographic characteristics

From the 384 distributed questionnaires there was a return rate of 86 %. Those 332 questionnaires were analyzed in this study. The mean age of the participants was 33 years (±7). 179 (53.9 %) were males and the majority of the participants were nurses 176 (53 %) followed by physicians 83 (25 %), and laboratory & pharmacy staffs 73 (22 %). The participants had a mean work experience of 8 (±7) years in the current hospital. Almost half of the participants 165 (49.7 %) have reported having a basic computer course in their study. Table 2 shows the detailed socio-demographic characteristics of the respondents.

**Table 2:** (Relative) frequencies of socio-demographic characteristics of the study participants including age, sex, work experience, professional category and IT course attendance

Socio-demographic characteristics	Absolute frequency	Relative frequency (%)
Age of respondents		
< 30	134	40.4 %
31-40	111	33.4 %
41-50	66	19.9 %
> 50	21	6.3 %
Sex		
Male	179	53.9 %
Female	153	46.1 %
Work experience in current hospital		
< 5 years	132	39.8 %
5-15 years	148	44.6 %
> 15 years	50	15.5 %
Professional category		
Physicians	83	25.0 %
Nurses	176	53.0 %
Lab&Pharmacists	73	22.0 %
IT course		
No IT course	98	29.5 %
Basic course	165	49.7 %
Advanced training	69	20.8 %

### Validation of the D&M constructs

The hypotheses were tested by inspecting the structural models and are summarized in Table 3, in which the standardized path coefficients and t-values are shown. They show the strength of the relationships between the independent and dependent variables. The results indicated that, with the exception of H7, all hypotheses proposed in this study were significant.

**Table 3:** Results of structural equation modeling in AMOS with the path coefficients for all of the 12 Hypotheses

Path	β	t-statistics	Supported?
System quality → EMR Use (*H1*)	0.32	3.12*	yes
System quality → User satisfaction (*H2*)	0.53	5.40**	yes
Information quality → EMR use (*H3*)	0.44	3.76*	yes
Information quality → User satisfaction (*H4*)	0.48	4.65**	yes
Service Quality → EMR Use (*H5*)	0.36	3.46*	yes
Service Quality → User satisfaction (*H6*)	0.56	7.26**	yes
EMR Use → User satisfaction (*H7*)	0.04	0.83	no
User satisfaction → EMR use (*H8*)	0.41	3.83*	yes
EMR Use → Perceived net-benefit (*H9*)	0.31	4.79**	yes
User satisfaction → Perceived net-benefit (*H10)*	0.60	8.22**	yes

Goodness of fit *χ* ^2^/*d.f.* = 2.39, GFI =0 .92, AGFI =0 .87, NFI = 0.92, RMSR = .056. * *p* < .05. *P* < 0.05,** < 0.01

The analysis showed that system quality has a significant influence on EMR use (β = 0.32, *P* < 0.05) and user satisfaction (β = 0.53, P < 0.01); information quality has significant influence on EMR use (β = 0.44, *P* < 0.05) and user satisfaction (β = 0.48, *P* < 0.01) and service quality has a strong significant influence on EMR use (β = 0.36, *P* < 0.05) and user satisfaction (β = 0.56, *P* < 0.01). User satisfaction has significant influence on EMR use (β = 0.41, *P* < 0.05) but the effect of EMR use on user satisfaction was not significant. Both EMR use and user satisfaction have significant influence on perceived net-benefit (β = 0.31, *P* < 0.01; β = 0.60, *P* < 0.01), respectively.

Service quality exhibited a stronger effect than system quality and information quality on both use and user satisfaction, which implies that service quality is an important factor in low-resource setting implementations. In terms of goodness of fit indicators, system quality, information quality and service quality accounted for 49.7 % of the variance in EMR use. Systems quality, information quality and service quality, accounted for 61.5 % of the variance in user satisfaction. EMR use and user satisfaction accounted for 72.8 % of the variance in perceived net-benefit.

### Mediating effect of computer literacy

The mediating effect of computer literacy with respect to EMR use and user satisfaction was measured using a method already applied by Nunes et al. [20]. In this procedure, a Student’s *t*-test was calculated for both variances and the comparison shows that computer literacy mediates the relationship between service quality and EMR use as well as user satisfaction. The result is shown in the following Table 4.

**Table 4:** Mediating effect of computer literacy in the relationship between service quality and EMR use as well as service quality and user satisfaction

Relationship	Moderator	β1	β2	t-stat	Supported?
Service quality → EMR use (*H11*)	Computer literacy	0.53	0.55	6.40*	yes
Service Quality → User satisfaction (*H12*)	Computer literacy	0.82	0.61	7.11**	yes

* *p* < .05. *P* < 0.05,** < 0.01

## Discussion

This study was conducted to empirically validate the generalizability of the D&M model by assessing the psychometric properties of the model in the context of low-resource setting hospitals. Additionally, insights were provided into HIS implementation success research by assessing the mediating effect of computer literacy on the relationship between service quality and EMR use as well as user satisfaction.

Our findings support all D&M constructs, except of the effect of EMR use on user satisfaction. The relationships of system quality, information quality, service quality, EMR use, user satisfaction and perceived net-benefit were proven to possess adequate psychometric properties and thus can be used as effective measures of EMR success in low resource settings. Individual relationships and their implication for practice are explained below.

System quality directly affects EMR use and user satisfaction (H1 and H2), thus implying that an increase in the quality of the system leads to an increase in EMR use and user satisfaction and hence, EMR success. System quality incorporates system ease of use, user friendliness, user interface and responsiveness. Thus, a net positive effect from these factors will result in a positive effect on EMR success. This result is consistent with different studies [7, 13, 16, 20] but different from e-government success [14] which might be attributed to the difference in user type and setting.

Information quality also positively affects EMR use and user satisfaction (H3 and H4), indicating that an increase in the quality of the information leads to an increase in user satisfaction and EMR use. The result is in line with previous studies [4, 20, 13, 14]. When the user’s attitude towards the information quality is more positive, the perceived usefulness of information will be higher. Therefore, while implementing EMR, managers should emphasize the following aspects: making sufficient information available, having good accuracy and ensuring on-time updating of information on the system and making sure that reports are in a format and layout health professionals routinely use and understand.

This study also shows that service quality of EMR implementation strongly influences EMR use and user satisfaction (H5 and H6). When users feel more satisfied with the service quality of the EMR, e.g. receiving good internal and external support, their satisfaction and probability of using the system will be higher. The result is consistent with other studies [7, 13, 16, 20]. Creating a supportive environment that is responsive to user concerns can increase the service quality. Additionally, it is necessary to provide more computers within the wards to allow clinicians entering patient data without having to wait for a free computer. Ensuring a sustainable power supply and giving immediate system support are also important factors to improve service quality. Given most implementations in those settings are donor supported, it is necessary for these organizations to provide satisfactory support to increase service quality and hence, user satisfaction and EMR use.

The findings of this study also indicate that the total effects of service quality on use and user satisfaction are substantially greater than those of system quality and information quality. This means that EMR implementation managers should pay much more attention to promoting the service quality of EMR systems. This result is not in line with other similar studies [7, 13, 16, 20] which is attributed to the setting differences. In low resource settings, there is a lack of computer access and sustainable power supply, which might make service quality a more determinant factor than the others.

The relationship between use and user satisfaction is a rarely researched factor in IS success literature [40]. In this study, we found that user satisfaction positively affects EMR use (H8) but our data does not support the hypothesis that EMR use positively affects user satisfaction (H7). This result might be attributed to the relatively small number of participants who currently use the system in the study hospitals.

EMR use and user satisfaction also positively affect net-benefit. This relationship is shown by many empirical studies and our result is consistent with them [11, 12, 40, 41]. Additionally, user satisfaction was found to be a stronger predicator of perceived net-benefit than EMR use. This result is also similar with other studies [14, 15, 42]. Assuming that user satisfaction is the user’s best estimate of the system capabilities, a stronger relationship between user satisfaction and perceived net-benefit is quite understandable.

The mediating effect of the computer literacy between service quality and EMR use as well as between service quality and user satisfaction was found to be significant. This result is consistent with Nunes et al. [20]. When we compare the two hypotheses, a stronger mediation effect was observed in the relationship between service quality and EMR use than with user satisfaction. This is a strong indicator that basic computer literacy is necessary for health professionals to increase their motivation of using the system. Therefore, during or before system implementation, in addition to specific user training, it is advisable to give additional basic computer courses to increase the success of the system.

### Strengths, weaknesses and generalizability of the study

Although we believe this study will make significant contributions to future EMR implementations in low-resource settings, some of the limitations must be noticed. First, the discussed findings were obtained from one single study from one EMR system in one country. Therefore it should be generalized to other populations with caution. However, with the context of the study, the survey result exhibited adequate validity and reliability. Second, our study is based on only self-reported questionnaires, which might have some response bias that, however, is almost unavoidable in cross sectional studies.

### Implications

To the level of our knowledge, this study is the first comprehensive validation of the D&M model to be applicable for EMR success evaluations in low resource settings. This study advances previous research by validating the model and testing the mediating effect of computer literacy in the service quality satisfaction relationship. The model provides not only an overall assessment of factors influencing EMR success but also the capability to identify the most problematic aspects of EMR implementation efforts. The magnitude of path coefficients provides useful insights into the relative importance of each subscale of the D&M model.

The validation of the D&M model under voluntary use and in low resource settings, is an input for and contribution to the IS scientific community, especially for the under-researched domain of healthcare ICT.

Practitioners should note that service quality was found to be the strongest determinant for EMR use and user satisfaction and user satisfaction was found to be the most important determinant factor for perceived net-benefit, hence EMR success. Therefore, managers should strive to improve hospital service quality and health professionals’ satisfaction. Additionally, the assessment of the computer literacy component in this study recognizes that the ability to use computers is an important aspect that influences an individual’s use of the implemented system in the hospital. Hence, this result implies that specific training to EMR is not enough for its success but the management should provide general basic computer courses to increase the system adoption.

### Future work

The instrument we used measures only perceived net-benefit but not actual net-benefit. Thus, future studies should carefully define the context in which net-benefit is measured and they should measure at user, hospital or governmental level (for example time savings in the clinical practice or return on investment). We also agree with D&M [12] that it is necessary to continuously challenge, validate and extend the proposed model under different user and implementation settings.

## Conclusion

The updated D&M model proved to be applicable to assess the EMR system success in low resource settings. Service quality was found to be the strongest determinant factor for EMR use and user satisfaction. User satisfaction was found to be the most important determinant factor for perceived net-benefit, hence, EMR success. Additionally, computer literacy was found to be a mediating factor between system quality and EMR use as well as between service quality and user satisfaction. Consequently, EMR implementers and managers in those settings should give priority in improving service quality of the hospitals like technical support and infrastructure; provide continuous basic computer trainings to health professionals; and give attention to the system and information quality of the system they want to implement.
